# Characterization of the Resistance and Force of a Carbon Nanotube/Metal Side Contact by Nanomanipulation

**DOI:** 10.1155/2017/5910734

**Published:** 2017-02-13

**Authors:** Ning Yu, Masahiro Nakajima, Qing Shi, Zhan Yang, Huaping Wang, Lining Sun, Qiang Huang, Toshio Fukuda

**Affiliations:** ^1^Intelligent Robotics Institute, Beijing Institute of Technology, Beijing 100081, China; ^2^Department of Micro-Nano Systems Engineering, Nagoya University, Nagoya 464-0814, Japan; ^3^Robotics and Microsystem Center, Soochow University, Suzhou 215006, China

## Abstract

A high contact resistance restricts the application of carbon nanotubes (CNTs) in fabrication of field-effect transistors (FETs). Thus, it is important to decrease the contact resistance and investigate the critical influence factors such as the contact length and contact force. This study uses nanomanipulation to characterize both the resistance and the force at a CNT/Au side-contact interface inside a scanning electron microscopy (SEM). Two-terminal CNT manipulation methods, and models for calculating the resistance and force at contact area, are proposed to guide the measurement experiments of a total resistance and a cantilever's elastic deformation. The experimental results suggest that the contact resistance of CNT/Au interface is large (189.5 kΩ) when the van der Waals force (282.1 nN) dominates the contact force at the interface. Electron-beam-induced deposition (EBID) is then carried out to decrease the contact resistance. After depositing seven EBID points, the resistance is decreased to 7.5 kΩ, and the force increases to 1339.8 nN at least. The resistance and force at the contact area where CNT was fixed exhibit a negative exponential correlation before and after EBID. The good agreement of this correlation with previous reports validates the proposed robotic system and methods for characterizing the contact resistance and force.

## 1. Introduction

The era of scaling silicon field-effect transistors (FETs) to ever-smaller dimensions is coming to a close. As an alternative to silicon, carbon nanotubes (CNTs) have received much interest because of their excellent mechanical [1, 2], electrical [3, 4], and thermal properties [5]. Many kinds of CNT-FETs have been fabricated, such as back-gated [6], top-gated [7], and gate-all-around [8]. However, high contact resistance between CNTs and drain/source metal electrodes limits the performance of CNT-FETs due to different work function. It is a challenge to decrease the contact resistance and to clarify the influence factors such as contact length and contact force at the interface.

The contact resistance was usually extracted from a total resistance between the CNTs and electrodes, rather than being directly measured experimentally. A typical measurement of total resistance [9] initially involved dispersing CNT solution on a Si wafer containing an electrode pair. Once CNTs bridged on the electrode pair, the total resistance across the two terminals was then measured. This method was easily carried out, but the likelihood of a CNT bridging the electrode pair was highly uncertain. Dielectrophoresis (DEP) technology improved the controllability of the CNT bridging process [10]. Bridging could be achieved using a tiny number of CNTs, and CNTs with specific properties could even be selected prior to assembly. However, the DEP process required many parameters to be regulated, such as the intensity, frequency, and duration of the applied electrostatic field [11, 12]. It was difficult to sufficiently control these parameters to achieve the specifically desired contact conditions, for example, a specific contact length. Micronanomanipulators have achieved a wide range of applications on electronic industry and biomedicine in recent decades, such as a unique microgripper with dual-axis force sensor [13] and nanomanipulators based on an Atomic Force Microscope (AFM) [14]. They are promising for good controllability of the targets. In our previous work, we successfully used nanorobotic manipulators inside a SEM to develop individual-CNT-based nanoposition sensors for the detection of approaching, touching, and sliding positions [15]. The controllability of nanorobotic manipulation provides an effective method to flexibly adjust the contact length of individual CNT.

For reducing the contact resistance, many techniques have been investigated. Joule heating was reported to be effective by Wang et al. [16]. They obtained a low ohmic contact resistance of 700 Ω between a multiwalled CNT (MWCNT) and tungsten (W) surface. Rapid thermal annealing at 600–800°C for 30 s was used by Lee et al. [17]. They obtained the contact resistance of 0.5–50 kΩ between a CNT and Au. Such progress was significant, but these techniques were limited by intense heat release at the contact junction, which leaded to poor control of the contact area/geometry. Focused ion-beam-induced deposition (IBID) could provide good spatial and time-domain control of the chemical vapor deposition of various materials [18]. It was used to form in situ contacts in CNT devices [19], and IBID with tungsten yielded an ohmic contact with CNTs [20, 21]. However, the focused ion beam damaged CNTs during long observation durations. Electron-beam-induced deposition (EBID) was similarly applied, but with minimal damage to the CNT. For example, Kim et al. used EBID with carbon to decrease the contact resistance [22], and Brintlinger et al. used EBID with gold to achieve a contact resistance of 10 kΩ [23].

Much progress has been made on measuring and decreasing the contact resistance. However, it is important to also investigate critical influence factors such as the metal work function and wettability, the contact length, or contact area, as well as the contact force. The effect of work function and wettability was studied by using fourteen different metals for CNT interconnections [24]. The effect of contact length was investigated by laser ablation with fixed CNT diameters [25] and by silicon-compatible test structures with a small range of CNT diameters [26]. As for the contact force, Greenwood and Williamson investigated the contact between nominally flat metal/metal surfaces [27], and experimental results implied a simple law *R* ∝ *F*^−0.9^, where *R* was the contact resistance and *F* was the contact force. Based on our previous study, the connection force between CNT/CNT junctions was confirmed to be composed of van der Waals force, EBID fixing force, and chemical bonding force, respectively [28, 29]. Furthermore, we quantized the van der Waals force at a CNT/metal end-contact interface by measuring a probe deflection [30]. However, a clear understanding of contact force at the CNT/metal side-contact interface still remains elusive. Additionally, to the best of our knowledge, there is no study to find its relationship with contact resistance.

In this paper, we present two-terminal methods to characterize the contact resistance at a CNT/metal side-contact interface and to investigate the contact force by using a nanorobotic system inside a field emission scanning electron microscopy (FE-SEM). This method allows CNTs to be individually manipulated, and its superiority in varying the arbitrary contact length in situ compared to the typical four-probe measurement [17, 31] shows a controllable CNT bridging process for the measurement of total resistance. EBID is carried out here to further control the contact area with tungsten deposits one by one and significantly decrease the contact resistance, which yields a strong CNT support with potential application on semiconductor nanodevices. Furthermore, the nanorobotic system is used to measure and analyze the force of a MWCNT/Au side-contact interface before and after EBID. It provides insight into the relationship between the contact force and the contact resistance.

## 2. System Configuration and Methods

A robotic system with two nanomanipulators was used here within a FE-SEM apparatus to measure a total resistance and characterize the force of a CNT/Au side-contact interface. Two-terminal CNT manipulation methods and calculation models were then developed to obtain the contact resistance and contact force.

### 2.1. System Configuration

**Figure 1:**
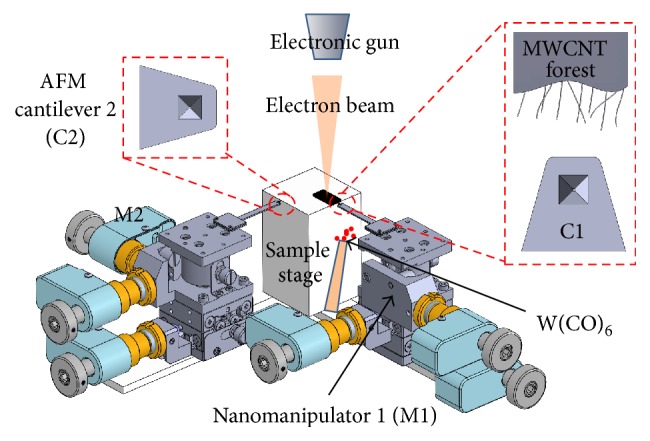
Schematic of the robotic system for characterizing the side contact between a CNT and metal electrodes inside a FE-SEM apparatus. M1 and M2 indicate manipulators 1 and 2, respectively. C1 and C2 indicate AFM cantilevers 1 and 2, respectively.

A two-terminal method was used to obtain the contact resistance at the CNT/Au interface, so the robotic system was configured with two manipulators based on a FE-SEM apparatus (JSM-6500F, JEOL), as shown schematically in Figure 1. Both of the manipulators were driven by picomotors (8301-UHV, Newport) in *x*-*y*-*z* directions with a resolution of 30 nm. Two AFM cantilevers covered with layers of Au (OMCL-TR400PB-1, OLYMPUS) were mounted on the manipulators as the end effectors. A MWCNT forest prepared by Arc charge method was placed on top of the sample stage. Tungsten hexacarbonyl (W(CO)_6_, MKBR3026V, SIGMA-ALDRICH) was introduced into the specimen chamber as the precursor for EBID with tungsten. A visual-based force feedback system was developed with the robotic system for real-time manipulation (not shown in Figure 1).

**Figure 2:**
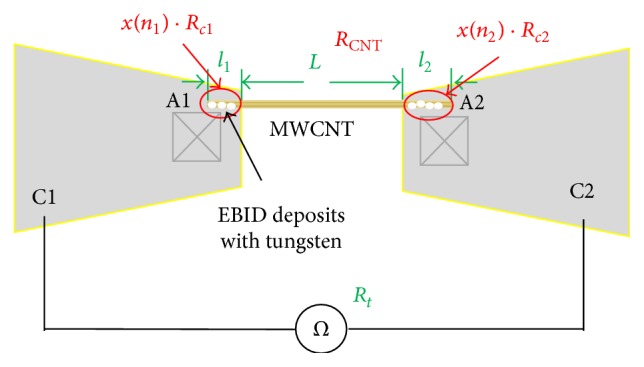
Schematic of the experimental setup used to measure the total resistance. *R*_*t*_ is the total resistance between cantilevers C1 and C2. *R*_*c*1_ and *R*_*c*2_ are the preliminary resistance of the CNT/Au side contact before EBID at contact areas 1 (A1) and 2 (A2), respectively. *x*(*n*_1_) and *x*(*n*_2_) are coefficients of the resistance variation before and after EBID; *n*_1_ and *n*_2_ are the numbers of EBID deposits at A1 and A2. *R*_CNT_ is the resistance of CNT. *l*_1_ and *l*_2_ are the contact lengths at A1 and A2. *L* is the gap length of the selected CNT.

**Figure 3:**
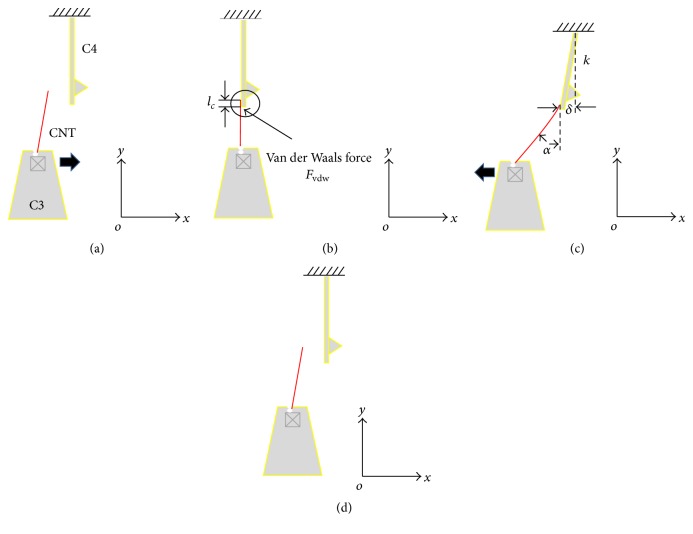
Schematic of the experimental setup used to measure the contact force. (a) CNT on cantilever 3 (C3) approaching cantilever 4 (C4). (b) The CNT attracted to the side of C4 with contact length *l*_*c*_. (c) Elastic deformation of C4. (d) CNT release and recovery of C4.

The AFM cantilevers were able to be mounted in different orientations to achieve different tasks. Horizontally fixing the two AFM cantilevers allowed the total resistance to be measured, as shown in Figure 2. Changing the orientation of one cantilever to vertical orientation allowed the contact force to be characterized, as shown in Figure 3(a).

### 2.2. Calculation Model of Contact Resistance

Figure 2 shows that a single CNT bridging two AFM cantilevers is used to obtain the side-contact resistance between the MWCNT and Au electrodes. The total resistance is measured between the two cantilevers by connecting with a source measure unit (Model 6430, Keithley) outside the FE-SEM apparatus.

To form the bridge with a single CNT, a CNT is initially picked up by manipulator 1 (M1) assisted by EBID [32]. This CNT is carried towards cantilever 2 (C2) on manipulator 2 (M2) and overlapped with cantilever C2 by some arbitrary length as we wish. As M1 slowly moves downward, sufficiently small distance between the CNT and cantilever C2 results in the CNT being attracted to the Au surface of cantilever C2 by van der Waals forces. The total resistance between the two cantilevers is then measured by Model 6430, Keithley. EBID with tungsten (W) can then be deposited at the CNT/Au interface to reduce the contact resistance at contact area 2 (A2).

As shown in Figure 2, the total resistance *R*_*t*_ between the two cantilevers is derived from the following equation:

(1) $$\begin{array}{c} R_{t}=x\left(\;n_{1}\;\right)\cdot R_{c1}+R_{\text{CNT}}+x\left(\;n_{2}\;\right)\cdot R_{c2}, \end{array}$$

where *R*_*c*1_ and *R*_*c*2_ are the preliminary resistances of the CNT/Au side contact before EBID at contact areas A1 and A2, respectively, *x*(*n*_1_) · *R*_*c*1_, and *x*(*n*_2_) · *R*_*c*2_ are the resistances at A1 and A2 after EBID, *x*(*n*_1_), and *x*(*n*_2_) are the coefficients of the resistance variation after EBID, *n*_1_ and *n*_2_ are the numbers of EBID deposits at A1 and A2, and *R*_CNT_ is the CNT resistance.

*R*_*t*_ is able to be obtained directly from the source measure unit. *x*(*n*_1_) · *R*_*c*1_ is a constant resistance, since the contact condition at A1 is constant as long as CNT is selected and picked up. In this case, we define *R*_*nc*1_ = *x*(*n*_1_) · *R*_*c*1_. *R*_CNT_ is equal to a product of the resistivity *r*_CNT_ and length *L*. *R*_*c*2_ is derived from dividing the preliminary contact resistivity before EBID (*ρ*_*c*2_) by the contact length between CNT and C2 (*l*_2_) [25]. After EBID with tungsten deposition, the resistance at A2 can be expressed by *x*(*n*_2_) · *R*_*c*2_. Specifically, *x*(*n*_2_) · *R*_*c*2_ is composed of the contact resistances of CNT/Au, Au/W, and W/CNT interfaces and the resistances of W deposits [33]. Despite an increased resistance caused by newly added contact resistances of Au/W and W/CNT interfaces and the resistances of W deposits, W deposition ensures electrical rigid contact benefitting for electron transport. As a result, the total resistance *x*(*n*_2_) · *R*_*c*2_ at A2 is decreased. *x*(*n*_2_) and *x*(*n*_2_) · *R*_*c*2_ can be changed by controlling *l*_2_ and *n*_2_. Thus, in the current study, the contact resistance refers only to that at A2.

Assuming that the CNT sample is defect-free and that *r*_CNT_ and *ρ*_*c*2_ are uniform along the CNT axis, then the following can be derived from (1):

(2) $$\begin{array}{c} R_{t}=R_{nc1}+r_{\text{CNT}}\cdot L+x\left(\;n_{2}\;\right)\cdot \frac{\rho_{c2}}{l_{2}}. \end{array}$$

When *n*_2_ = 0, arbitrarily changing *l*_2_ by controlling M1 or M2 will lead to different values of *R*_*t*_. Analyzing *R*_*t*_ under different situations yields *ρ*_*c*2_ and *R*_*c*2_ before EBID. Furthermore, *x*(*n*_2_) and *x*(*n*_2_) · *R*_*c*2_ after EBID can be calculated by analyzing different values of *R*_*t*_ measured with increasing *n*_2_ when *l*_2_ is fixed.

### 2.3. Calculation Model of Contact Force

The mechanical and electrical contact condition is affected by the applied contact force. Therefore, monitoring the force of the CNT/Au contact is necessary. Based on the method of measuring the total resistance in Section 2.2, the force of the CNT/Au side contact includes contributions from two kinds of force. One is the attractive van der Waals force at the CNT/Au interface before EBID. The other force is the fixing force of EBID deposits. Electrostatic forces are omitted since the CNT and AFM cantilevers are well grounded.

To quantify the contact force, elastic deformation of cantilever 4 (C4) is intended to measure, which is derived from the two kinds of force at the CNT/Au interface. The principle and manipulation strategy are shown schematically in Figure 3. The robotic system still contains two cantilevers C3 and C4. C3 is horizontally bounded on M1, while C4 is vertically orientated on M2, shown in Figure 3(a). C3 is used to pick up a single MWCNT from the CNT forest. The picked CNT is then allowed to contact with the side of C4 by van der Waals forces (*F*_vdw_), as shown in Figure 3(b). After good contact, C3 is moved in the *x*-axis, and C4 thereby experiences an elastic deformation *δ*, as shown in Figure 3(c). With increasing of *δ*, the elastic restoring force on C4 becomes larger than *F*_vdw_, and it leads to the CNT release and C4 in situ recovery, as shown in Figure 3(d).

Obviously, cantilever C4 is acted by two forces: one is the van der Waals force at the CNT/Au contact interface, and the other is the elastic resilience force resulting from elastic deformation. According to the law of force balance, the van der Waals force per unit contact length (*f*_vdw_) is obtained as follows:

(3) $$\begin{array}{c} f_{\text{vdw}}=\frac{F_{\text{vdw}}}{l_{c}}=\frac{k\delta_{\max}}{l_{c}}, \end{array}$$

where *F*_vdw_ is the van der Waals force, *l*_*c*_ is the CNT/Au contact length on C4, *k* is the spring constant of C4, and *δ*_max_ is the maximum deformation at the moment of CNT release.

Similarly, when EBID deposits are formed at the CNT/Au interface on C4, the total force (*F*_*t*_) including contributions from *F*_vdw_ and a fixing force of EBID (*F*_EBID_) is expressed as

(4) $$\begin{array}{c} F_{t}=F_{\text{vdw}}+F_{\text{EBID}}=k\delta_{\max}. \end{array}$$

The fixing force per EBID deposit (*f*_EBID_) is

(5) $$\begin{array}{c} f_{\text{EBID}}=\frac{k\delta_{\max}-F_{\text{vdw}}}{n}, \end{array}$$

where *n* is the number of EBID deposits.

## 3. Experimental Results and Discussion

In this section, the total resistance was measured with varying the contact length (*l*_2_) and increasing the number of EBID deposits (*n*_2_). The contact resistance at A2 was calculated by analyzing these measured total resistances using the above proposed calculation model. The van der Waals force and the EBID fixing force at the CNT/Au interface were also investigated by measuring the deformation of C4.

### 3.1. Experimental Materials

**Figure 4:**
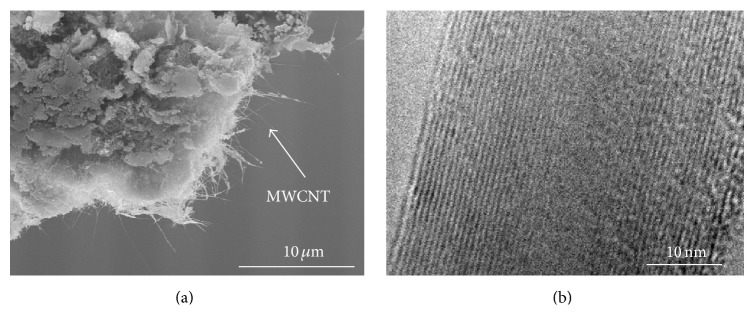
The MWCNT samples. (a) SEM image. (b) TEM image.

The MWCNT forest used in these experiments was synthetized by Arc charge method. Their length was ~20 *μ*m, and the diameter was 20–50 nm.  Figure 4 shows the SEM and TEM images of these MWCNT samples.

Each cantilever chip (OMCL-TR400PB-1) consisted of two different length levels. The 100 and 200 *μ*m-long level had spring constant of 0.09 and 0.02 N/m, respectively. The Au coating thickness was 40–50 nm.

### 3.2. Experimental Conditions

1. The SEM chamber pressure was approximately 10^−4^ Pa.
2. The beam current was 0.01 nA.
3. The acceleration voltage was 10 kV.
4. The mean deposited length per minute of a EBID point was 37 nm/min when using W(CO)_6_, and its maximum diameter was about 100 nm.

### 3.3. Results of Contact Resistance

**Figure 5:**
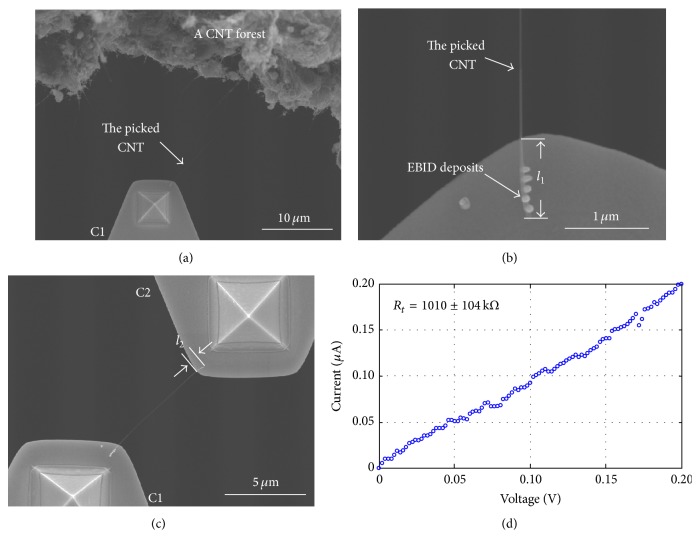
Measurement of total resistance of the CNT/Au side contact before EBID. (a) CNT selection and pick-up. (b) Magnification of the picked CNT and EBID deposits on C1. (c) Bridging of the CNT to C2. (d) Measured *I*-*V* curve from which total resistance was obtained.

A longer, straight CNT was targeted from the CNT forest. Cantilever C1 was controlled to contact the CNT fixed by EBID and then to pick the CNT up. This process was shown in Figures 5(a) and 5(b). The diameter of the picked CNT was 28 nm, and the contact length *l*_1_ was 940 nm. Five EBID points were deposited, each with deposition time of 3 min. Then, we bridged the selected CNT to C2 with a contact length *l*_2_ of 0.43 *μ*m, as shown in Figure 5(c). The contact length was a visually determined length after we confirmed the CNT was firmly contacted with the Au surface at A2. After bridging the CNT, electron beam was turned off to avoid the irradiation on the electrical contact of CNT/Au interface [33], and three minutes later, we measured the total resistance of the two cantilevers by using the source measure unit. The unit generated a sweep voltage, and a PC recorded the current passing through the CNT. To avoid unwanted heating effects, the voltage was constrained to 0–0.2 V, and its step size was 0.002 V.  Figure 5(d) showed the measured *I*-*V* curve, from which an average total resistance of 1010 ± 104 kΩ was obtained. After this test, electron beam was turned on, and C1 was moved such that the CNT was released from C2 and then allowed to form a new contact with a contact length *l*_2_. The total resistance was then measured once more. This procedure was carried out four times. In the four tests, each value of contact length *l*_2_ yielded a different total resistance, respectively, whereas the CNT resistivity *r*_CNT_ and the contact resistivity *ρ*_*c*2_ were constants. Based on (2), we could obtain these parameters as listed in Table 1. Table 1 showed that a larger contact length resulted in a lower contact resistance. The contact length of 1.34 *μ*m resulted in a contact resistance of 189.5 kΩ. However, this result was still much higher than ideal value. Theoretically, the contact resistance is governed by the quantum limit, and in the case of ideal contacts, it is 6.45 kΩ accounting for two conduction channels per CNT shell [9]. EBID technique was then applied to decrease the resistance.

**Figure 6:**
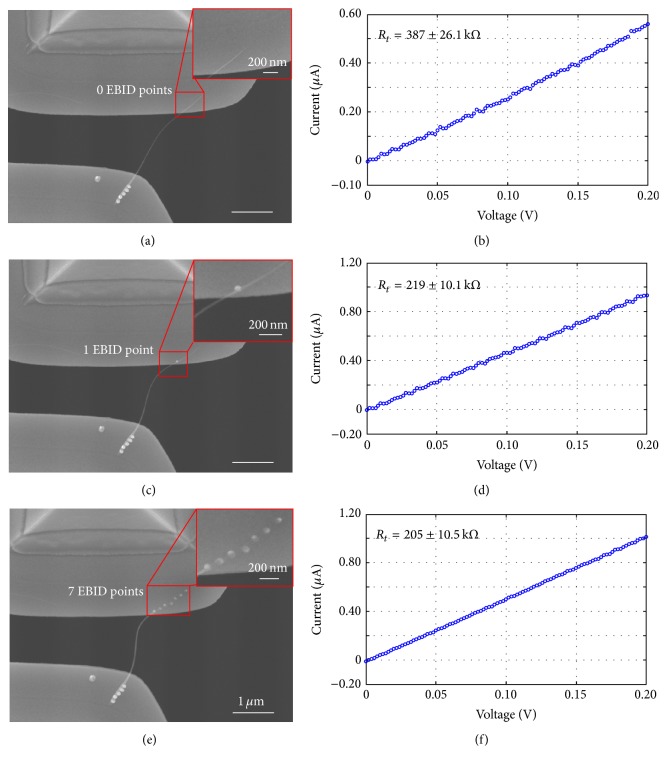
Measurement of total resistances with increasing number of EBID deposits. (a), (c), and (e) show SEM images of the bridged CNT with 0, 1, and 7 EBID deposits at A2, respectively. (b), (d), and (f) show the corresponding *I*-*V* curves recorded by a Keithley 6430 source measure unit. Scale bar indicates 1 *μ*m.

Fixing the contact length *l*_2_ at 1.34 *μ*m, EBID points (each with a 3 min deposition time) were deposited on the CNT/Au interface at A2. The number of deposited points ranged from one to seven, and the corresponding total resistance was measured with electron beam being off after each EBID deposit. The experimental results were shown in Figure 6. Since images with increasing the number of EBID points were very similar, images from 2–6 EBID points were not shown in Figure 6. The *I*-*V* curves in Figures 6(b), 6(d), and 6(f) were linear, indicating an ohmic contact in each test. Total resistances were obtained from these *I*-*V* curves and were listed in Table 2. Resistances after EBID at A2 were then calculated using (2), and a contact resistance of 7.5 kΩ was obtained finally after seven EBID deposits, which was in good agreement with the theoretical value and previously reported experimental values demonstrated in [17, 23]. Compared with the preliminary contact resistance of 189.5 kΩ (*n* = 0), the resistance at A2 was decreased by 96.0% (*x*_(*n*2)_ = 4.0).

### 3.4. Results of Contact Force

**Figure 7:**
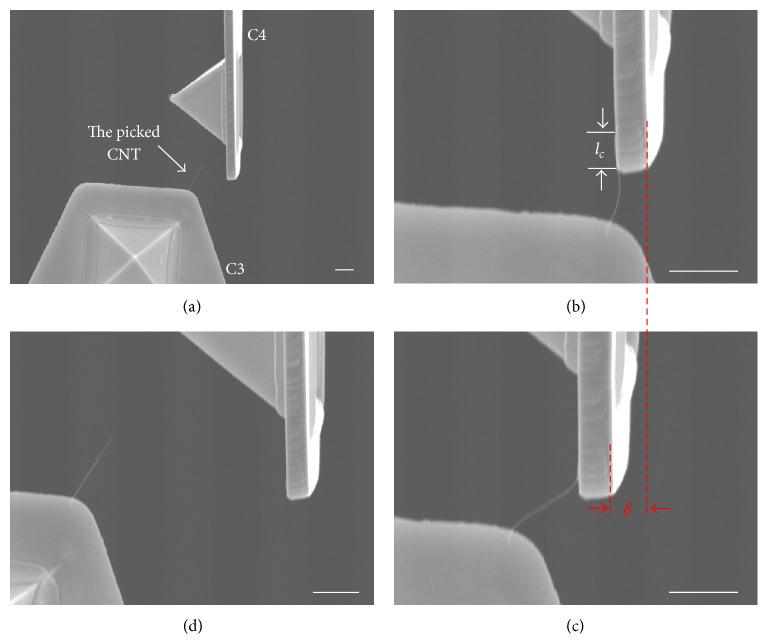
Measurement of van der Waals force at the CNT/Au interface. (a) A picked CNT approaching cantilever C4. (b) Attracted contact of CNT/Au side surface with C4. (c) Coordinated motion of C4 following C3. (d) CNT release from C4. Scale bar indicates 1 *μ*m.

We first tested the van der Waals force *F*_vdw_ of a CNT and Au side contact before EBID in this section. After a single MWCNT (outer diameter of 25 nm) pick-up by C3, it was moved to gradually approach C4 in a vertical orientation, as shown in Figure 7(a). The CNT then attractively contacts the side of the Au surface by *F*_vdw_ with contact length *l*_*c*_ shown in Figure 7(b), and M1 began to move to the left. C4 also moved following C3 and generated an elastic deformation *δ* shown in Figure 7(c). Finally, the CNT was released when the van der Waals force became less than the recovery force of C4, as shown in Figure 7(d). A video recording of this process was used to measure *δ*_max⁡_. Four measurements of *δ*_max⁡_ were carried out and the resulting data were summarized in Table 3. Using (3), the van der Waals force per unit contact length (*f*_vdw_) was then calculated. The *f*_vdw_ values for the four *δ*_max⁡_ measurements were 208.9, 209.7, 222.4, and 200.8 nN/*μ*m, and the average was 210.5 nN/*μ*m. To verify this result, a theoretical value was calculated from $f_{\text{vdw}}=H\sqrt{D}/16d^{5/2}$, where Hamaker constant *H* was 12.46 × 10^−20^ J [34], outer diameter of the CNT *D* was 25 nm, and the distance between the CNT and Au surface was assumed to be 0.5 nm. The calculated result *f*_vdw_ was 220.1 nN/*μ*m, which was comparable to the above experimental result. This validates the proposed method for measuring the force of the CNT/Au contact.

**Figure 8:**
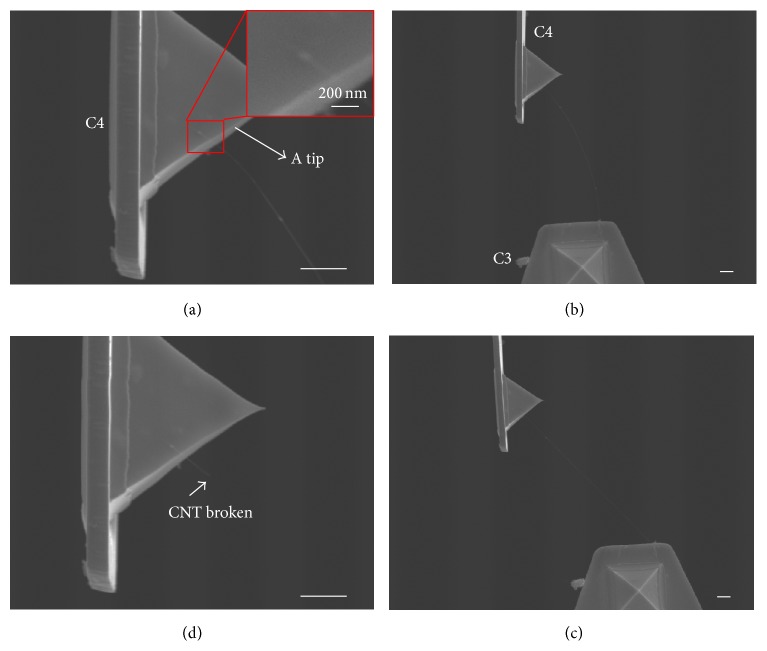
Measurement of EBID fixing force. (a) A picked CNT bridging to the tip of cantilever C4 with one EBID point from cantilever C3. (b) The original state of the two cantilevers after CNT bridging. (c) Coordinated motion of C4 following C3. (d) Stretched CNT broken in the middle and C4 recovered. Scale bar indicates 1 *μ*m.

The total force with one EBID deposit was measured similarly. A picked CNT from cantilever C3 was bridged to the pyramidal tip of cantilever C4, as shown in Figure 8(a). One EBID point was then deposited on the interface to fix the CNT. The original state of the two cantilevers after CNT bridging was shown in Figure 8(b). Cantilever C3 was moved and formed a resulting deformation at C4 in Figure 8(c). The CNT was under tension in this process and finally broke in the middle in Figure 8(d) when its stress limit was surpassed. Four tests were carried out with recording of the experimental data and the measured *δ*_max⁡_ in Table 4, in which test 1 and test 2 used the same CNT sample, and tests 3 and 4 used two further samples. Additionally, contact length in test 2 was approximated to be zero in Table 4 because only the CNT end-point touched the Au surface of the cantilever. All of the CNT samples were broken in the experiment, so we could only estimate the value of the total contact force within a range instead of obtaining an exact value. Actually, the contact force after EBID was strong enough to enhance the electrical rigid contact at CNT/Au interface and to decrease the resistance by improving the electron transport. Considering the cantilever tip angle of 70°, the total force in these four tests was more than 96.2, 151.1, 183.1, and 179.4 nN, respectively. The resulting *f*_EBID_ values were calculated using (5) and were more than 66.7, 151.1, 130.5, and 80.5 nN, respectively.

The EBID that used to decrease contact resistance in this study not merely well controlled the size of the contact area but also increased the contact force. The contact area and contact force were closely related to the size and shape of EBID deposits, which were in turn influenced by many factors such as the electron beam energy and the location of incidence. This study focused on investigating a valid method for measuring the contact resistance and contact force and the relationship between them. Thus, all of the factors affecting the size and shape of EBID deposits were kept constant in these experiments. In terms of this point, the *f*_EBID_ values for the four tests should in theory have been comparable. The larger deviation in these values was caused by the different break positions and varying tensile stresses of the CNT samples. We concluded that *f*_EBID_ was >151.1 nN.

Additionally, based on our previous work, the fixing force at the CNT/Au contact area was increased with more than 30 s electron irradiation when the contact area was magnified to be 30,000 times [30]. Therefore, all of the tests on the force measurement in this study were carried out with a smaller magnification (less than 10,000 times). In case of larger magnification for confirming the contact condition between CNT and Au surface, the observation usually took less than 30 s to reduce the electron irradiation as much as possible.

### 3.5. Relationship between the Resistance and the Force of the CNT/Au Side Contact

**Figure 9:**
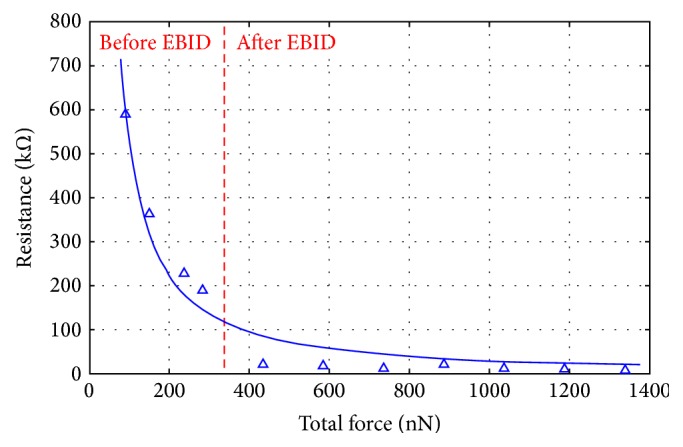
Relationship between the force and the resistance of the CNT/Au contact before and after EBID. Hollow triangles represent the contact resistance under the different contact force, whereas the solid line represents a fitting of these experimental data.

Table 1 showed the contact resistance for different contact lengths before EBID, in which the van der Waals force per unit contact length (*f*_vdw_) was 210.5 nN/*μ*m. Thus, the contact force before EBID (i.e., the van der Waals force) in the four tests was calculated to be 90.5, 147.4, 235.8, and 282.1 nN. Furthermore, since *f*_EBID_ was >151.1 nN, the total force at the CNT/Au contact area after seven EBID deposits was >1339.8 nN. Other cases are summarized in Table 5. The contact resistance *x*(*n*_2_) · *R*_*c*2_ and the force *F*_*t*_ before/after EBID in Table 5 were fitted to a very simple proportion *x*(*n*_2_) · *R*_*c*2_ ∝ *F*_*t*_^−1.26^, as shown in Figure 9. From Figure 9, the contact resistance became smaller with an increased contact force. This was explained by the fact that the contact resistance is inversely proportional to the contact area, while the contact area becomes larger with the increased contact force. Additionally, Figure 9 was obtained by setting *f*_EBID_ = 151.1 nN for the convenience of fitting. This setting affected the fitting index of *F*_*t*_, and *x*(*n*_2_) · *R*_*c*2_ ∝ *F*_*t*_^−1.15^ would be obtained if *f*_EBID_ was set to 300 nN. Increasing *f*_EBID_ to 1500 nN yielded *x*(*n*_2_) · *R*_*c*2_ ∝ *F*_*t*_^−1.00^. Thus, the contact resistance was negative-exponentially related to the contact force. This negative-exponentially relationship was similar to the reported *R* ∝ *F*^−0.90^ [27] and *R* ∝ *F*^−0.94^ [35], which proved the validity of our method.

## 4. Conclusions

This paper reported a nanorobotic system containing two manipulators within a SEM apparatus. This system allowed both the contact resistance and the force between a MWCNT and Au-coated cantilevers to be measured before and after EBID. Experimental results showed a contact resistance of 189.5 kΩ before EBID and a decreased resistance of 7.5 kΩ after 7 EBID deposits. Contact force at the CNT/Au interface before EBID was measured to be 210.5 nN/*μ*m and increased by >151.1 nN per EBID point. Fitting the experimental data yielded a negative exponential relationship between the resistance and force of the contact area, and good agreement of the relationship with previous reports validated the proposed method. In future, our robotic system will be expanded to investigate the contact between CNTs and other metals and to fabricate nanodevices such as CNT-FETs in combination with EBID.

## Figures and Tables

**Table 1:** The total resistance and contact resistance before EBID.

Tests	Contact length *l*_2_ (*μ*m)	Total resistance *R*_*t*_ (kΩ)	Contact resistivity *ρ*_*c*2_ (kΩ·*μ*m)	Contact resistance *R*_*c*2_ (kΩ)
1	0.43	1010 ± 104	254.0	590.7
2	0.70	755 ± 35.4	254.0	362.9
3	1.12	598 ± 26.7	254.0	226.8
4	1.34	387 ± 26.1	254.0	189.5

**Table 2:** The total resistance and the resistance at A2 after EBID.

Number of EBID points *n*_2_	The total resistance *R*_*t*_ (kΩ)	The resistance at A2 *x*(*n*_2_) · *R*_*c*2_ (kΩ)	*x*(*n*_2_) (%)
0	387 ± 26.1	189.5	100
1	219 ± 10.1	21.5	11.3
2	215 ± 6.6	17.5	9.2
3	209 ± 2.5	11.5	6.1
4	216 ± 9.0	18.5	9.8
5	209 ± 8.5	11.5	6.1
6	208 ± 17.0	10.5	5.5
7	205 ± 10.5	7.5	4.0

**Table 3:** Measurement of the van der Waals force at the CNT/Au interface before EBID.

Tests	Contact length *l*_*c*_ (*μ*m)	Spring constant *k* (N/m)	Deformation *δ*_max_ (*μ*m)	Van der Waals force per unit contact length *f*_vdw_ (nN/*μ*m)
1	0.18	0.02	1.88	208.9
2	0.29	0.02	3.04	209.7
3	0.49	0.02	5.45	222.4
4	1.08	0.09	2.41	200.8

**Table 4:** Measurement of EBID fixing force at the CNT/Au interface.

Tests	1	2	3	4
Number of EBID points	1	1	1	1
Contact length *l*_*c*_ (*μ*m)	0.14	≈0	0.25	0.47
Spring constant *k* (N/m)	0.02	0.09	0.09	0.09
Deformation *δ*_max_ (*μ*m)	14.06	4.91	5.95	5.83
*kδ*_max_ (nN)	281.2	441.9	535.5	524.7
Total contact force (nN)	>96.2	>151.1	>183.1	>179.4
Calculated *F*_vdw_ (nN)	29.5	≈0	52.6	98.9
The fixing force of EBID *f*_EBID_ (nN)	>66.7	>151.1	>130.5	>80.5

**Table 5:** Resistance and force of the CNT/Au contact before and after EBID.

Tests	Contact length *l*_2_ (*μ*m)	Number of EBID deposits	Resistance at A2 (kΩ)	The force *F*_*t*_ before EBID (nN)	Total force *F*_*t*_ after EBID (nN)
1	0.43	0	590.7	90.5	
2	0.70	0	362.9	147.4	
3	1.12	0	226.8	235.8	
4	1.34	0	189.5	282.1	
5	1.34	1	21.5		>433.2
6	1.34	2	17.5		>584.3
7	1.34	3	11.5		>735.4
8	1.34	4	18.5		>886.5
9	1.34	5	11.5		>1037.6
10	1.34	6	10.5		>1188.7
11	1.34	7	7.5		>1339.8
